# Activity-based costing of health-care delivery, Haiti

**DOI:** 10.2471/BLT.17.198663

**Published:** 2017-11-16

**Authors:** Ryan K McBain, Gregory Jerome, Fernet Leandre, Micaela Browning, Jonathan Warsh, Mahek Shah, Bipin Mistry, Peterson Abnis I Faure, Claire Pierre, Anna P Fang, Jean Claude Mugunga, Gary Gottlieb, Joseph Rhatigan, Robert Kaplan

**Affiliations:** a Partners In Health, 800 Boylston Street, Suite 1400, Boston, Massachusetts, United States of America (USA).; b Zanmi Lasante, Port-au-Prince, Haiti.; c Harvard Business School, Boston, USA.; d Department of Global Health and Social Medicine, Harvard Medical School, Boston, USA.; e Analysis Group, Inc., Boston, USA.

## Abstract

**Objective:**

To evaluate the implementation of a time-driven activity-based costing analysis at five community health facilities in Haiti.

**Methods:**

Together with stakeholders, the project team decided that health-care providers should enter start and end times of the patient encounter in every fifth patient’s medical dossier. We trained one data collector per facility, who manually entered the time recordings and patient characteristics in a database and submitted the data to a cloud-based data warehouse each week. We calculated the capacity cost per minute for each resource used. An automated web-based platform multiplied reported time with capacity cost rate and provided the information to health-facilities administrators.

**Findings:**

Between March 2014 and June 2015, the project tracked the clinical services for 7162 outpatients. The cost of care for specific conditions varied widely across the five facilities, due to heterogeneity in staffing and resources. For example, the average cost of a first antenatal-care visit ranged from 6.87 United States dollars (US$) at a low-level facility to US$ 25.06 at a high-level facility. Within facilities, we observed similarly variation in costs, due to factors such as patient comorbidities, patient arrival time, stocking of supplies at facilities and type of visit.

**Conclusion:**

Time-driven activity-based costing can be implemented in low-resource settings to guide resource allocation decisions. However, the extent to which this information will drive observable changes at patient, provider and institutional levels depends on several contextual factors, including budget constraints, management, policies and the political economy in which the health system is situated.

## Résumé

**Objectif:**

Évaluer la réalisation d'une analyse des coûts par prestation et par durée dans cinq établissements de soins communautaires en Haïti.

**Méthodes:**

En collaboration avec les parties prenantes, l'équipe du projet a décidé que les prestataires de soins devraient indiquer l'heure de début et de fin de la consultation dans le dossier médical d'un patient sur cinq. Nous avons formé un responsable du recueil des données par établissement, qui a saisi manuellement les horaires ainsi que les caractéristiques des patients dans une base de données et a enregistré ces dernières dans un entrepôt de données en ligne chaque semaine. Nous avons calculé les coûts de capacité par minute pour chaque ressource utilisée. Les durées indiquées ont été multipliées par le coût de capacité unitaire à l'aide d'une plate-forme virtuelle automatisée, par laquelle ces informations ont été transmises aux administrateurs des établissements de soins.

**Résultats:**

Entre mars 2014 et juin 2015, le projet a suivi les services cliniques dispensés à 7162 patients externes. Le coût des soins prodigués pour différentes affections était très variable entre les cinq établissements, en raison de l'hétérogénéité du personnel et des ressources. Par exemple, le coût moyen d'une première consultation prénatale allait de 6,87 dollars des États-Unis ($ US) dans un établissement de premier niveau à 25,06 $ US dans un établissement de haut niveau. Au sein des établissements, nous avons également observé des variations de coûts, qui étaient dues à des facteurs tels que les comorbidités des patients, le moment d'arrivée des patients, les stocks de fournitures dans les établissements et le type de consultation.

**Conclusion:**

Il est possible de déterminer les coûts par prestation et par durée dans les structures à faibles ressources afin d'orienter les décisions concernant l'affectation des ressources. Cependant, la mesure dans laquelle ces informations vont entraîner des changements observables au niveau des patients, des prestataires et des institutions dépend de plusieurs facteurs contextuels, notamment des contraintes budgétaires, de la gestion, des politiques et de l'économie politique dans laquelle se trouve le système de santé.

## Resumen

**Objetivo:**

Evaluar la implementación de un análisis de gastos basado en el tiempo invertido por actividad en cinco centros sanitarios comunitarios en Haití.

**Métodos:**

Junto a las partes interesadas, el equipo del proyecto decidió que los profesionales sanitarios debían introducir los horarios del comienzo y el final de la consulta con el paciente en el dossier médico de uno de cada cinco pacientes. Formamos a un recopilador de datos por cada centro, que introdujo manualmente los registros de tiempo y las características de los pacientes en una base de datos, los cuales enviaba a un almacén de datos en la nube cada semana. Calculamos el coste de capacidad por minuto para cada recurso utilizado. Una plataforma automatizada en línea multiplicó el tiempo registrado por la tasa del coste de capacidad y proporcionó la información a los administradores de los centros sanitarios.

**Resultados:**

Entre marzo de 2014 y junio de 2015, el proyecto registró los servicios clínicos a 7162 pacientes. El coste de la atención para condiciones específicas variaba considerablemente entre los cinco centros, debido a la heterogeneidad del personal y de los recursos. Por ejemplo, el coste medio de una primera consulta de atención prenatal variaba de 6,87 USD en un centro de bajo nivel a 25,06 USD en un centro de alto nivel. En los centros, observamos una variación similar en los costes, debido a factores como las comorbilidades de los pacientes, el tiempo de llegada del paciente, el almacenamiento de suministros en los centros y el tipo de consulta.

**Conclusión:**

Los gastos basados en el tiempo invertido por actividad se pueden implementar en entornos con pocos recursos para orientar la toma de decisiones sobre asignación de recursos. Sin embargo, la medida en que esta información generará cambios notables a nivel de los pacientes, profesionales e instituciones depende de varios factores contextuales, incluidas las limitaciones presupuestarias, la gestión, las políticas y la economía política en que se encuentra el sistema de salud.

## ملخص

**الغرض:**

تقييم طريقة تطبيق التحليل الموجه بالوقت الخاص بتقدير التكاليف المعتمدة على الأنشطة في خمس منشآت صحية مجتمعية في هايتي.

**الطريقة:**

قرر فريق المشروع بالتعاون مع الجهات المعنية أنه يجب على مقدمي خدمات الرعاية الصحية كتابة أوقات البدء والانتهاء من استقبال المريض في كل خمسة ملفات طبية للمريض. ولقد قمنا بتدريب شخص واحد ليجمع البيانات في كل منشأة، وهو الذي يقوم بكتابة تسجيلات الوقت وخصائص المريض في قاعدة للبيانات ثم يقوم بتسليم البيانات إلى مستودع البيانات القائم على التخزين السحابي أسبوعيًا. لقد قمنا بحساب تكلفة الاستيعاب في الدقيقة لكل مورد مستخدم. قام النظام الآلي المستند إلى شبكة الإنترنت بالجمع بين الوقت المكتوب مع نسبة تكلفة الاستيعاب ثم قدم المعلومات إلى مديري المنشآت الصحية.

**النتائج:**

قام المشروع في الفترة ما بين مارس/آذار من عام 2014 ويونيو/حزيران من عام 2015 بتتبع الخدمات المقدمة لـ 7162 من المرضى الخاضعين للعلاج في عيادات خارجية. وقد تباينت تكلفة الرعاية لبعض الحالات المحددة بشكلٍ واسع عبر خمس منشآت، نظرًا لتواجد عنصر عدم التجانس في مستوى توظيف طواقم العمل والموارد. على سبيل المثال، تراوح متوسط تكلفة الزيارة الأولى الخاصة برعاية الحوامل من 6.87 دولار أمريكي في منشأة منخفضة المستوى إلى 25.06 دولارًا أمريكيًا في منشأة عالية المستوى. ولقد لاحظنا وجود تباين مماثل في التكاليف داخل المنشآت، بسبب بعض العوامل مثل الاعتلالات المشتركة للمريض، ووقت وصول المريض، وتجهيز الإمدادات الطبية في المنشآت، ونوع الزيارة.

**الاستنتاج:**

يمكن تطبيق نظام التكلفة الموجهة بالوقت والمعتمدة على الأنشطة في الأماكن قليلة الموارد بهدف التأثير على قرارات تخصيص الموارد. ومع ذلك، إن الحد الذي من خلاله ستؤدي هذه المعلومات إلى إحداث تغييرات ملحوظة على مستويات المريض، ومقدم الخدمة الصحية، والمؤسسات يعتمد على عدة عوامل سياقية، حيث تشمل قيود الميزانية، والإدارة، والسياسات، والاقتصاد السياسي الذي يتعامل به النظام الصحي. ****

## 摘要

**目的:**

旨在评估海地五个社区医疗机构实施估时作业成本法分析的情况。

**方法:**

项目团队与利益相关者共同决定，医疗护理提供方应该在每五名患者的医疗档案中输入接触患者的开始时间和结束时间。我们在每个机构内培训一名数据采集人员，该名人员负责在数据库中手动输入时间记录和患者特征并每周将数据提交到基于云的数据仓库中。对于采用的各项资源，我们计算每分钟的经营成本。由基于网络的自动化平台将经营成本率乘以报告的时间，并向医疗机构的管理人员提供该信息。

**结果:**

项目追踪了 2014 年 3 月到 2015 年 6 月间的 7162 位门诊患者的临床服务。由于人员和资源的不均衡性，这五个机构针对特定病症的护理成本相差很大。例如：第一次产前检查的平均费用由低级机构的 6.87 美元到高级机构的 25.06 美元不等。由于患者并存病、患者到达时间、机构内的库存物资以及就诊类型的不同，我们在机构内观察到了类似的成本差异。

**结论:**

估时作业成本法可在资源贫乏的情况下指导资源分配决策。然而，该信息将在多大程度上推动患者、提供方和机构层面发生明显的变化取决于预算限制、管理、政策和医疗系统所处政治经济背景等环境因素。

## Резюме

**Цель:**

Оценить реализацию анализа расчета себестоимости по видам деятельности на основе фактора времени в пяти общинных медицинских учреждениях в Гаити.

**Методы:**

Совместно с заинтересованными сторонами проектная группа решила, что поставщики медицинских услуг должны вносить время начала и окончания приема пациента в медицинское досье каждого пятого пациента. Для каждого медицинского учреждения мы подготовили по одному сборщику данных, который вручную вводил в базу данных записи о времени приема и характеристиках пациентов и каждую неделю отправлял эти данные в облачное хранилище. Мы рассчитали себестоимость одной минуты для каждого используемого ресурса. Автоматизированная веб-платформа умножила указанное в записях время на величину себестоимости и предоставила информацию администраторам медицинских учреждений.

**Результаты:**

В период с марта 2014 года по июнь 2015 года проект отслеживал оказание клинических услуг для 7162 амбулаторных пациентов. Себестоимость медицинского обслуживания для конкретных условий сильно варьировалась среди пяти медицинских учреждений из-за неоднородности кадровых и материальных ресурсов. Например, средняя себестоимость первого посещения женской консультации для дородового наблюдения варьировалась от 6,87 долл. США в медицинском учреждении низкого уровня до 25,06 долл. США в медицинском учреждении высокого уровня. На уровне медицинских учреждений мы наблюдали аналогичную вариацию себестоимости из-за таких факторов, как сопутствующие заболевания пациентов, время поступления пациентов, запасы лекарственных препаратов в медицинских учреждениях и тип посещения.

**Вывод:**

Расчет себестоимости по видам деятельности на основе фактора времени может быть реализован в условиях ограниченности ресурсов для рационального распределения ресурсов. Тем не менее степень, в которой эта информация приведет к наблюдаемым изменениям на уровне пациентов, поставщиков и медицинских учреждений, зависит от нескольких контекстуальных факторов, включая бюджетные ограничения, управление, стратегии и политико-экономическую ситуацию, в которой находится система здравоохранения.

## Introduction

Understanding the cost of health-care delivery is essential for guiding resource allocation decisions that have direct implications for patient care and health outcomes.^1^ Optimizing resources available for patients is particularly important in low-resource settings. In these settings, tough decisions are unavoidable as human and financial resources allocated to one particular programme necessarily, if implicitly, reduces the availability of resources for other programmes.

Health ministries need valid information on condition-specific costs and patient outcomes to make informed resource allocation decisions and cost–benefit trade-offs. For example, robust evidence exists that human immunodeficiency virus (HIV) health-care delivery by nurses and lay workers, rather than doctors, is a cost-saving mechanism that improves access to treatment and with only minimal adverse effects on patient outcomes.^2^ A range of other, broader cost–benefit trade-offs is also inherent in many health policy decisions. Such as, to reach more people, policy-makers may prioritize urban over rural populations,^3^ emphasize curative over preventative care^4^ and focus on infectious rather than noncommunicable diseases.^5^

Traditional costing approaches typically measure costs at the departmental level through top-down allocation procedures. They do not provide accurate patient-level cost information and are not based on service delivery processes.^6^ For example, the management accounting system for hospitals,^7^ allocates aggregated expenditures to cost centres such as transportation, information technology, equipment and security. These costs, in turn, are distributed to medical services such as women’s health, pharmacy and radiology, with unit costs estimated by dividing service-level costs by the number of patients seen or service units delivered.

This approach is adequate for understanding programmatic costs and major cost drivers, and to calculate an average cost per patient or per service.^8^^,^^9^ However, it fails to capture whether, how and why clinical processes, activities and protocols vary from one patient to another, including among patients who present with the same condition.^10^ Nor does the approach give information about the actual mix of resources used to treat individual patients. Traditional cost methods simplistically assume homogeneity across patients and providers. However, evidence indicates that clinical care is highly idiosyncratic and that variation can sometimes serve a purpose, such as to customize care for a patient’s comorbidities and medical history.^11^ Equally important, such methods do not link practice variations to variation in patient outcomes. Such information is critical for informing the hospital administration about staffing and delivery of day-to-day health-care services.

**Fig. 1:**
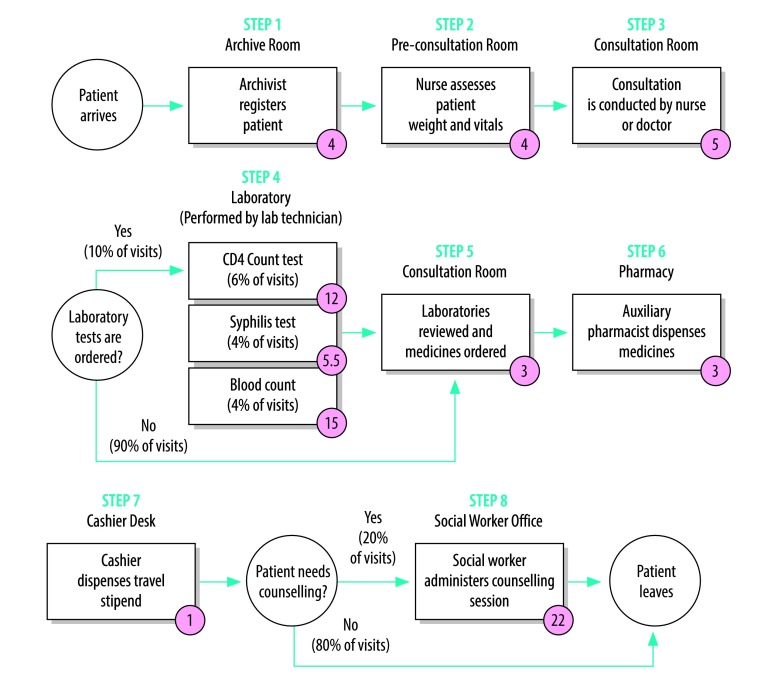
Process map of routine HIV care at Lacolline health facility, Haiti

To reduce variation in resource use that does not contribute to patient outcomes, the time-driven activity-based costing approach takes the patient, not a clinical department, as the unit of analysis.^12^ The approach enables hospital administrators to understand the total costs of all the resources used for patient care. The cost data inform process improvement, staffing and other resource allocation decisions, ultimately leading to improved patient outcomes.^13^^,^^14^ By following the resources used, the approach provides a detailed breakdown of each clinical activity, including the variation in time from one patient to the next and the use of specific personnel, equipment, supplies and space at each step of the care cycle.^15^ To display the actual service experiences, data collectors inductively construct process maps from the resources used. Fig. 1 presents an illustrative process map for a routine HIV-patient visit at Lacolline, a primary-level health facility in Haiti.

HIV: human immunodeficiency virus.

Note: Process map is based on 1219 observed patients. Green circles represent average time in minutes for each step.

The study implementated time-driven activity-based costing analysis in five outpatient departments of health-care facilities in Haiti, conducted by Partners In Health and Zanmi Lasante. This analysis was done to inform resource allocation decisions, provide a basis for cost–effectiveness analysis and consider novel financing mechanisms for broadening access to health care.

## Methods

### Local setting

Haiti is a low-income country and has an average annual gross domestic product (GDP) per capita of 818 United States dollars (US$).^16^ The health ministry’s operational budget is less than 2% of GDP^17^ and, typically, poor residents pay over a third of their health expenditures out of their pockets.^18^

Since the 2010 earthquake, where more than 150 000 Haitians lost their lives,^19^ Haiti’s health system has been severely constrained. Not only did morbidity and mortality levels increase by the earthquake, the subsequent displacement of approximately 1.5 million Haitians also made these individuals vulnerable to opportunistic infections and susceptible to malnutrition.^20^ Shortly after the earthquake, a wide-scale cholera epidemic erupted, which continues to present day.^21^

Partners In Health and its sister organization in Haiti, Zanmi Lasante, is one of the largest health-care providers in the country. Partners In Health collaborates with the health ministry to deliver health-care services to over 1.2 million Haitians living in the Central Plateau and Lower Artibonite, just to the north of Port-au-Prince.

### Set-up

Partners In Health, in collaboration with the local health ministry, proposed to conduct time-driven activity-based costing analysis at five health facilities throughout its provider network, ranging from a primary-health centre with no inpatient ward to a district-level hospital with over 50 beds. The facilities were located from the country’s eastern border to the west coast. This enabled the project to examine whether cost variation could be attributed to geographic and cultural diversity. Because Partners In Health works in particularly rural areas of the country, we conducted the approach knowing that survey instruments could be adapted for a similar cost exercise in urban areas like Port-au-Prince, using local translation and simple didactic tools. However, some of the implications of the findings may vary as a function of these differences. For example, in rural areas the supply chain is weaker and access to care is worse,^22^ which may increase patient incentive to seek care at local traditional healers in such areas.

Partners In Health selected to study a set of outpatient services based on two criteria. First, the service should be high-volume so that the project would capture the preponderance of care delivered by the organization. Second, the service should be high-priority for the organization, which included treatment of conditions prevalent among vulnerable populations.^23^ We investigated nine clinical services: HIV care, tuberculosis care, antenatal care, women’s health visits, acute care visits for adults and separately for children, noncommunicable diseases, malaria treatment and family planning. These services represented over three-quarters of the care provided.

### Implementation

The project team implemented the costing activities in five steps. First, we engaged with stakeholders to minimize the disruption of services for the already under-resourced health facilities. The team held a series of meetings with administrators of Zanmi Lasante to determine which data collection methods would produce high-quality information with the least disruption and with limited staff and training. We relied on simplified paper-based time-driven activity-based costing form that the team had modified to capture only essential information for analysis. The costing form included information on patient characteristics, patient arrival time, patient wait time, patient consultation time, primary diagnosis, medicines prescribed and laboratories ordered time stamps for consultation times.

The project team decided that the approach could capture a random cross-section of the patient population by having data clerks insert a costing form in every fifth patient’s medical dossier on which the health-care provider would enter the information required. In return, the team promised that hospital administrations would receive key deliverables to inform and improve facility activities. These deliverables included process maps of each service, the amount of time, human and financial resources committed for clinical activities, the frequency of medicines and laboratory tests ordered for each conditions. Team members and appointed clinical staff at facilities also provided hospital administrations with result-based recommendations for improvements. This process also encouraged local collaboration, input and local ownership.

Second, we trained people involved in data collection. We held one-week training for all data collectors at Partners In Health’s headquarters in Port-au-Prince. Data collectors comprised of local Haitian staff within Zanmi Lasante, task shifted to perform activities from other clinical research projects approaching completion, as well as two new hires. Salaries were supported by grant-based funding for the duration of the project. We informed the data collectors about the data collection tools, including the costing form inserted into medical dossiers and a facility survey, which data collectors used to catalogue equipment and medical supplies throughout the facilities. Their responsibilities entailed entering data from these forms into an electronic database and submitting forms for review on a weekly basis. Separately, research staff held a one-hour session with administrators at each facility to inform about the project’s objectives and which part of the costing form the project team expected the health-care providers to complete.

Third, we tried to ensure a high quality of the collected data. Given the importance of accurate time stamps in executing the method, the project purchased digital clocks to be placed in locations throughout the five health facilities. The project team held a two-hour group meeting at each facility with health-care providers, laboratory and pharmacy staff to familiarize them to the data collection entry process. We placed a data collector at each facility to gather and screen the costing forms at the end of the day, who then could provide immediate feedback to any staff member who had entered any information incorrectly. Separately, data collectors recorded additional information concerning equipment, room dimensions and administrative staffing. Lastly, independent of patient-level information, a project manager interviewed departmental heads at the beginning of implementation to obtain supplemental inputs for the costing analysis. This included operational information, such as the number of hours different types of providers worked and overall staffing levels for provider types. Additionally, to ensure appropriate understanding of tasks, a project manager visited the health facilities on a weekly basis.

Fourth, data collectors manually entred all gathered information into a Partners In Health database each week and uploaded the data to an open source cloud-based data warehouse, by using CommCare (Dimagi, Cambridge, United States of America).^24^ Research staff located outside Haiti accessed CommCare to review and analyse all data on a monthly basis. Analysis of data included identification of outliers and inconsistencies in data entry.

Fifth, to estimate cost for our nine selected outpatient services, we first calculated the capacity cost rate for each type of resource, personnel, equipment and space, used during a health-care visit. We did this by dividing the annual cost of each resource by the total number of minutes that the resource could be used per year. To obtain the total cost of the continuum of care for a patient, an automated system multiplied the electronically reported minutes for each resource used with its associated calculated capacity cost rate and presented it as a total sum of the patient visit.^12^ We used an R script (R Foundation for Statistical Computing, Vienna, Austria) to automate the data analysis and a Shiny data visualization tool (RStudio®, Boston, USA). In addition, we used descriptive and inferential statistics for inputs, such as characteristics of the patient population and variation in wait time, by condition, by facility. To adjust for significant fluctuation in currency conversion from US$ to Haitian Gourdes, we used a composite average of live exchange rates between March 2015 and June 2016, valued at US$ 1.00 to 56.63 Haitian Gourdes.^25^ We combined final cost estimates with process maps for individual health conditions and services and shared these with hospital administrations. The data visualization for all nine outpatient services across the five facilities are available at: https://htdata.pih-emr.org/dhis/shiny/.

For patients presented with multiple complaints, we separated services that were inherently standardized, such as a routine HIV visit, from services that were more heterogeneous in presentation during the analysis and interpretation phase. For the latter, we combined conditions into higher-level clinical pathways, such as integrated management of childhood illness for acute conditions.

## Results

From March 2014 to June 2015, we collected data from 7162 patients, with at least 1000 unique observations per facility (Table 1). The sample size enabled us to analyse variations in time allocations and resources at the patient-, service-, and facility-level.

**Table 1:** Number of patients observed at each health facility, Haiti, March 2014 to June 2015

Outpatient service	Health facility, no. of patients observed (%)				
	Belladere (*n* = 1114)	Boucan Carre (*n* = 1456)	Hôpital St Nicholas (*n* = 1251)	Hinche (*n* = 1511)	Lacolline (*n* = 1830)
**HIV care**	85 (7.6)	240 (16.5)	0 (0.0)	743 (49.2)	395 (21.6)
**Tuberculosis care**	11 (1.0)	32 (2.2)	0 (0.0)	57 (3.8)	27 (1.5)
**Antenatal care**	146 (13.1)	152 (10.4)	550 (44.0)	0 (0.0)	441 (24.1)
**Women’s health**	36 (3.2)	13 (0.9)	15 (1.2)	0 (0.0)	124 (6.8)
**Acute care**					
For adults^a^	232 (20.8)	349 (24.0)	99 (7.9)	286 (18.9)	250 (13.7)
For children^b^	217 (19.5)	205 (14.1)	183 (14.6)	24 (1.6)	160 (8.7)
**Noncommunicable diseases**	208 (18.7)	167 (11.5)	84 (6.7)	45 (3.0)	120 (6.6)
**Malaria treatment**	32 (2.9)	5 (0.3)	18 (1.4)	16 (1.1)	8 (0.4)
**Family planning**	9 (0.8)	45 (3.1)	45 (3.6)	15 (1.0)	103 (5.6)
**Other**	138 (12.4)	248 (17.0)	257 (20.9)	325 (21.5)	202 (11.0)

HIV: human immunodeficiency virus.

^a^ A patient 18 or older was considered an adult.

^b^ A patient younger than 18 years was considered a child.

Note: We tracked patient’s care cycle by measuring the time each patient spent at each step of the cycle.

**Fig. 2:**
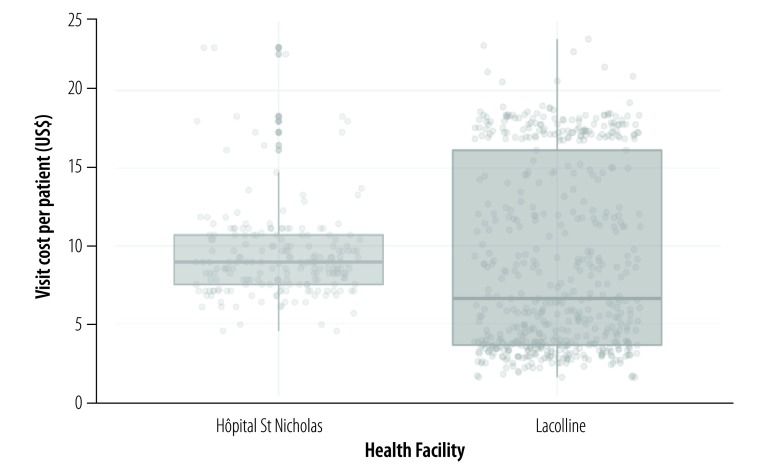
Patient-level cost variation in a women’s health outpatient visit at two health-care facilities, Haiti, 2015

The cost of care for specific conditions varied widely across the five facilities, due to heterogeneity in staffing and resources. For example, the average cost of a first antenatal-care visit ranged from US$ 6.87 at a low-level facility (Boucan Carre) to US$ 25.06 at a high-level facility (Hinche). Fig. 2 shows the patient-level costs for women’s health visits at two facilities.

US$: United States dollars.

Note: Total sample is 741 patients, 436 patients from Lacolline and 305 from Hôpital St Nicholas. Each dot on the box plots represents a patient’s cost associated with a visit. Whiskers represent the interquartile range across all patients.

**Fig. 3:**
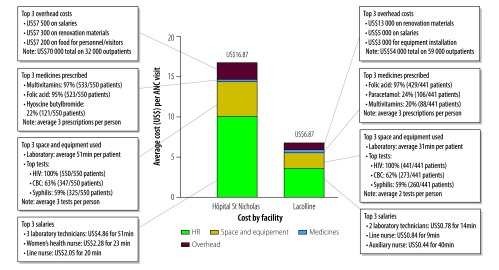
Cost allocations for antenatal care visits at two health-care facilities, Haiti, 2015

ANC: antenatal care; CBC: complete blood count; HIV: human immunodeficiency virus; HR: human resources; US$: United States dollars.

Note: Data are from 550 visits at Hôpital St Nicholas and 441 visits at Lacolline.

To show an example how to identify specific sources of variation in the roles that staff performed, how long they spent performing these roles and how often they prescribed medicines and ordered laboratory tests, we present the patient costs for antenatal care at two facilities (Fig. 3). We found that the higher-level facility, Hôpital St Nicholas, had more staffing and time dedicated to processing laboratory work than the lower-level facility Lacolline. Hôpital St Nicholas also had highly-trained staff providing educational sessions to newly-pregnant women. Additionally, we found that health-care providers prescribed folic acid to more than 90% of the pregnant women at both facilities (429 out of 441 pregnant women received a prescription in Lacolline and 523 out of 550 in Hôpital St Nicholas). However, health-care providers at Hôpital St Nicholas prescribed much more multivitamins to pregnant women than providers at Lacolline (97%; 533/550 versus 20%; 88/441, respectively). This finding allowed for Boston-based staff to make adjustments in the supply chain for transporting medicines to Haiti.

We performed a wide range of comparisons at macro- and micro-levels. At a macro-level, for example, we found that the ratio of health-care providers to patients ranged from 1:331 in at Hôpital St Nicholas, versus 1:1261 in Boucan Carre with the average consult time being 11 minutes at Hôpital St Nicholas versus 5 minutes at Boucan Carre. At a micro-level, we found that within facilities for specific conditions, average wait time, length of consultation and number of laboratory tests ordered varied as a function of patient arrival time. Patients who arrived earlier in the day waited longer, but received longer consultations and had more laboratory tests ordered.

## Discussion

We found that lay health workers in low-resource settings can effectively implement the rigorous time-driven activity-based costing approach when they are supplied with appropriate orientation, training, resources and tools. Staff members were able to examine variations in process maps across facilities, for the same services and health conditions. Such comparisons allowed for identification of alternative, cost-saving practices. Including for example, having patients return to their physician to review laboratory results on the same day of their initial visit cost less than having patients return the next day. An additional benefit of the approach was its capacity to project costs over time, based on redesigned clinical protocols, increased staffing and improved stock of medicines, as well as under scenarios, in which universal health coverage is met. Such as, demographic trends in population growth suggested that greater demand for antenatal care services will occur over the next several years.^26^ The approach informed how staffing, infrastructure and supplies would need to be adjusted to accommodate the higher future service demands.

Understanding and managing costs in resource-poor settings like Haiti is of importance, given the scarcity of financial and clinical resources available for treating the populations. Partners In Health envisions using insights from this approach to introduce process efficiencies and optimize the staffing of its many facilities around the world, and the organization has already begun the process in Malawi and Rwanda. The organization will also use the approach to describe the return on investment of additional resourcing from governments and external funders. For instance, we recently incorporated the cost estimates from time-driven activity-based costing, in return on investment analyses presented to the Haitian President and health ministry and the estimates are now being used to inform decision-making on prioritized health conditions at a national level.^27^ Historically, public health facilities in Haiti have struggled to institute a unified framework of cost recovery with the support of the central ministry, due to the heterogeneity in quality of care and type of service provision across facilities. Time-driven activity-based costing has acted to solidify a framework around which Partners In Health and the ministry can discuss provision of care in relation to costs.

Variation in care across sites highlighted several opportunities for improvement. We found for example, that certain facilities were under-prescribing medicines, not because health-care providers were unaware of the appropriate medicines and treatment regimens, but because of stock-outs. Solving this problem requires a more holistic restructuring of the health-service delivery system, such as supply chain management, to effectively address the primary causes.^28^^,^^29^ Likewise, we found that short consultation times at several facilities were not the result of desirable process efficiencies, but indicators of low-quality processes associated with a shortage of human resources. This was of particular concern, as a growing body of evidence indicates that the simple and inexpensive act of talking more with patients leads to better outcomes and lower total costs.^30^

Our results indicate that in settings of extreme resource scarcity, exercises like time-driven activity-based costing underscore the dearth of options available for system-level improvement.^12^ This approach may serve to demonstrate what it would cost to achieve improved care and coverage and to leverage this information, to advocate for new investments by the international community.

We faced some logistic difficulties when implementing the costing approach. First, as found in other contexts,^31^ consistent data collection and entry proved challenging, particularly in large facilities, due to the complex patient flow. In smaller health centres, patient tracking was more transparent and easier. Additionally, geographic barriers and poor internet connectivity delayed feedback loops at smaller, remote facilities. Despite these setbacks, overall fidelity to the protocol was high, due to supervisory staffing on the project. Also the 20% sampling rate yielded a large sample size for reliable data analysis.

A limitation to the approach was the heterogeneity in patient populations across the five facilities. Routine HIV care at a district-level hospital was likely treating a different group of patients, for example, those with complications, as compared to those seen at a lower-level facility. Data collection forms could have been modified to account for this. Additionally, we examined only outpatient services. While this represented the bulk of patient care, the data collection did not capture the more complex care delivered at higher-level facilities. An extension of this work might examine high-volume, high-cost inpatient services at the larger facilities as done in a previous Partners In Health project in Haiti.^32^ Lastly, patient-level costs were not connected to patient-level outcomes. While this is a challenge even in high-income settings, longitudinal tracking of patients would have provided insights into the ways in which variations in services, and costs, were associated with outcomes.

The time-driven activity-based costing approach provides useful information with diverse applications. We believe that the information presented herein could offer a reference point to other service delivery organizations and health facilities implementing the approach. At the patient-level, the approach has the capacity to offer insights about sources of cost and clinical service variation, which in turn can enhance the quality of service delivery and improve resource allocation. In Haiti, the approach provides a foundation for the government to evaluate costs and clinical services across the country, for both outpatient and inpatient care.
